# RocHealthData.org: Development and usage of a publicly available, geographic source of social determinants of health data

**DOI:** 10.1017/cts.2024.484

**Published:** 2024-02-15

**Authors:** Kathleen D. Holt, Gretchen Roman, Laura McIntosh, Jamie Kleinsorge, Jeanne Holden-Wiltse, Nancy M. Bennett

**Affiliations:** 1 Clinical and Translational Science Institute, University of Rochester, Rochester, NY, USA; 2 Center for Community Health and Prevention, University of Rochester, Rochester, NY, USA; 3 Department of Family Medicine, University of Rochester, Rochester, NY, USA; 4 Center for Applied Research and Engagement Systems, University of Missouri, Columbia, MO, USA; 5 Department of Medicine, School of Medicine and Dentistry, Rochester, NY, USA

**Keywords:** Data curation, data accessibility, geographic information systems (gis), social determinants of health, website design

## Abstract

Access to local, population specific, and timely data is vital in understanding factors that impact population health. The impact of place (neighborhood, census tract, and city) is particularly important in understanding the Social Determinants of Health. The University of Rochester Medical Center’s Clinical and Translational Science Institute created the web-based tool RocHealthData.org to provide access to thousands of geographically displayed publicly available health-related datasets. The site has also hosted a variety of locally curated datasets (eg., COVID-19 vaccination rates and community-derived health indicators), helping set community priorities and impacting outcomes. Usage statistics (available through Google Analytics) show returning visitors with a lower bounce rate (leaving a site after a single page access) and spent longer at the site than new visitors. Of the currently registered 1033 users, 51.7% were from within our host university, 20.1% were from another educational institution, and 28.2% identified as community members. Our assessments indicate that these data are useful and valued across a variety of domains. Continuing site improvement depends on new sources of locally relevant data, as well as increased usage of data beyond our local region.

## Introduction

Social determinants of health (SDoH) are defined as elements of a person’s physical or social environment, and include constructs such as neighborhood resources, population characteristics, geography, and aspects of the physical environment. Insights from these data can be vital in ascertaining the impact on public health [1]. These SDoH variables have been widely recognized as important, yet sometimes under-reported, predictors of disease prevalence and severity, as well as poor health outcomes. A sampling of just a few of the many articles on this topic shows the importance of SDoH in understanding disease rates, courses of non-melanoma skin cancer, and type 2 diabetes, as well as the general importance of SDoH in health risk prediction [2–4].

Factors that may influence the under-utilization of health information, including SDoH, are the difficulty locating, harmonizing, and accessing these data across platforms and sources [5–7]. A recent report from the U.S. Department of Health and Human Services notes the many challenges in obtaining social determinants data and recommends the development of tools and platforms that allow for easier access [8]. The use of Geographic Information Systems (GIS) to map information is one such tool that could be more widely used to understand factors impacting population health [9]. Making SDoH data available and accessible to a wide variety of users, within a GIS framework, can lead to greater understanding of the impact of SDoH [10–12].

**Figure 1.**
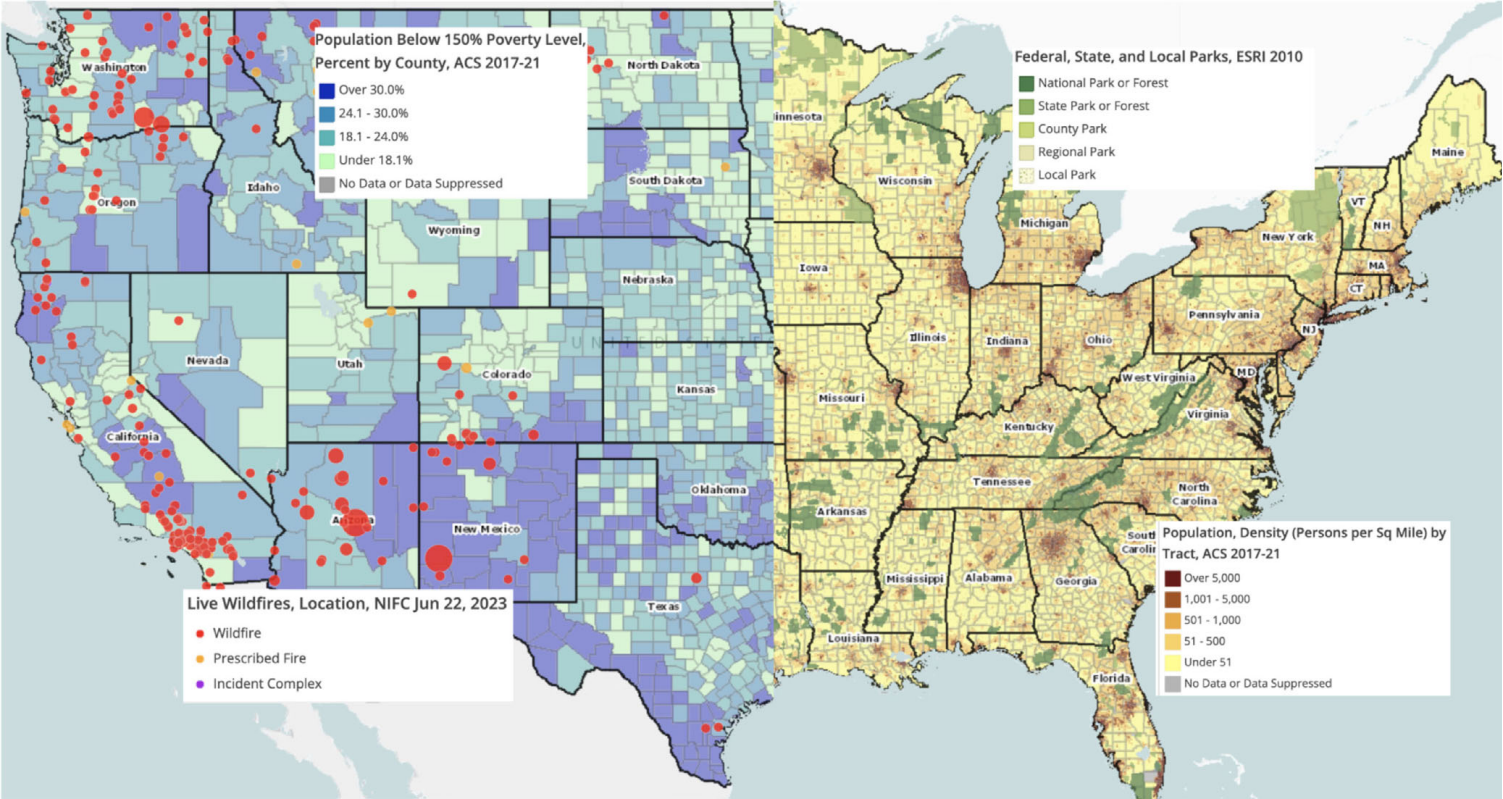
Examples of data available at RocHealthData.org: American Community Survey data on population density and poverty; National Interagency Fire Center data on recent wildfires; Environmental Systems Research Institute data on US Parklands.

To help address the challenges of having a widely available, easily accessible, and unified source of SDoH data, the University of Rochester Medical Center’s Clinical and Translational Science Institute (URMC CTSI) contracted in 2018 with The University of Missouri Center for Applied Research and Engagement Systems (CARES) to create a flexible web-based tool (RocHealthData.org) to allow access to thousands of publicly available national datasets. These datasets include SDoH elements such as poverty, climate events, population density, physical environment, crime, food access, access to care, and health outcomes, and are available in report formats (as Community Health Needs Assessment reports described below) as well as in mapped form. Figure 1 shows a few of these elements, mapped as they appear within the RocHealthData.org site, zoomed in to certain locations and in a variety of geographic granularities (these locations were selected from across the US to highlight the variability of data within the geographies).

Data availability at the site is informed by more than a decade of applied research. In 2012, CARES worked alongside the National Committee on Vital Statistics, several nonprofit hospital systems, and public health organizations to develop a core set of data to help decision-makers and health providers better understand the social, economic, and environmental conditions impacting population health in their service areas. This core set of indicators was used by nonprofit hospital partners to meet IRS community benefit requirements and develop tri-annual Community Health Needs Assessments (CHNA). The current CHNA database hosted by CARES provides more than 300 indicators from 121 secondary data sources, available at county, zip code, and census-tract level geographies. The RocHealthData.org local health assessment pulls from the CARES CHNA database to visualize health outcomes and social determinants of health within a regional health framework.

Many other websites offer free access to health-related data, and exploration using geographic mapping: Healthdata.org provides access to global burden of disease health data; OurWorldInData.org curates longitudinal data based on global trends impacting living conditions; UDSMapper.org facilitates the evaluation of the federally funded Health Center Program. While all these sites allow access to publicly available geographically based data, RocHealthData.org focuses on publicly available data to support regional population health initiatives. CARES has created a regional CHNA report that includes indicators benchmarked to state and national averages, which are available at a variety of geographies (eg., city, ZIP code, census tract, school district, and legislative district), and include demographic stratifications (where available) for age, race, gender, and ethnicity [13]. Indicators in the RocHealthData.org local health assessment are visualized in a variety of ways (eg, tables, dials, maps, and graphs) for greater ease of understanding and to facilitate longitudinal comparisons.

A pivotal piece of the RocHealthData (RHD) site is our ability to share information about newly available data. The site hosts several sets of local data (periodically or continuously updated), including community health indicators [14], COVID-19 vaccination rates by census tract [15], and reports of local respiratory illness prevalence, hospitalizations, and deaths [16]. These local sets of data contain various levels of geographic specificity (region, county, and census tract) as well as details by demographic groupings (sex, race, and ethnicity). Through our blog posts and periodic updates, we highlight these data in conjunction with local community organizations [17,18] and seasonal or environmental events [19,20].

We inform users about these sets of data through emails, blog posts, and periodic updates, as well as geocoding and mapping of user-supplied data. The URMC CTSI offers this tool, free of charge, to any interested users. Registration is required, and registration includes access to all available data. The site was revamped in late 2019 based on user feedback (which included requests for enhanced site navigation) and changes in the underlying data management structures at CARES. All national data sets and sources are updated by CARES and available at the site when released by the data sources (eg., Census Department, USDA, etc). Governance of the site at URMC CTSI is facilitated by our Population Health Data Group, a steering committee that meets semimonthly to review site usage, available data, and plans for future content. Decisions as to the availability of future data are driven by research interests as well as community health needs, as this group consists of both University and regional community-based organization representatives

To encourage use and membership on the site, in-person and virtual training opportunities (highlighting of available datasets, functionality of mapping, and report capabilities) were offered when the website was relaunched in 2019. These training sessions were held for members of the University community, regional community-based organizations, and our steering committee. Subsequently, brief tutorial training videos were created by CARES and are available on the site for users’ reference.

The URMC Center of Community Health and Prevention and CTSI developed this site to foster data accessibility within our community. The intent of this paper (Special Communication) is to describe the success of this initiative: how access to SDoH data has impacted local health policy, the high level of engagement of our users, and information on visitor use of and behavior at the site.

## Methods

As per URMC’s Guidelines for Determining Human Subject Research, this project did not meet the definition of research according to the U.S. Department of Health and Human Services regulation 45 CFR 46, and thus was not required to undergo review from the Research Subject Review Board.

To help describe the scope, use, and effectiveness of the RHD site, we examined information from a variety of sources, including qualitative feedback, visitor data through web analytics, and descriptive data on registered users.

Qualitative feedback was gathered via personal communication with several community organizations over the last three years and was elicited by an open-ended item on the website asking, “What kinds of information would you like to see highlighted or presented at RocHealthData.org?”

Data from Google Analytics for the time period January 1, 2020 through May 31, 2023 was also used to assess user engagement with and behavior at the website. A free service launched by Google [21] in November 2005, Google Analytics (GA) allows website administrators to collect visitor data through web analytics and has been used to assess a wide variety of topics, such as developing marketing strategies [22] and facilitating academic library usage [23]. We used several common industry standard metrics [24,25] to describe user engagement with the site in 4 areas: visitor type (new and returning), sessions (number and duration per visitor and visitor type), page views (total, including repeated views of a single page and by visitor type), and bounce rate (single page session with no additional site interaction or other pages visited, by visitor type).

A final source of information about the site’s usage comes from detailed data on our registered users. As a step in the registration process, registrants are required to provide an email address and an organizational affiliation (University of Rochester, Other Educational or Community Organization, or Community Member), and asked to provide zip code (personal or workplace).

We used the straightforward nature of qualitative description to establish a thematic framework matrix for qualitative data analysis. Two study team members (GR and KDH) worked separately to paraphrase important issues in the feedback and worked together to triangulate these into relevant topic areas, themes, and exemplar quotes [26].

Descriptive statistics were used to analyze quantitative data, including mean, frequency, and duration (Google Analytics, Google, Mountainview, CA and Excel, Microsoft Corporation, Redmond, WA). Finally, quantitative and qualitative data were integrated to accentuate any positive or negative findings [27].

## Results

### Qualitative feedback

Personal communication about the site was largely positive from a variety of community organizations and from individuals with varying roles within those organizations (Table 1). In addition to mentioning their generally use of the website and general favorable impressions, respondents also shared how particular data were used to improve specific projects.

**Table 1.** Qualitative feedback from RocHealthData (RHD) visitors

Topic area	Theme	Exemplar quote
Use of particular data to improve specific projects	COVID-19 vaccination rates	“From a public policy perspective, the vaccination rates by census tract are phenomenal [physician within our health system].”
		“We are actively using this, almost daily, to help us prioritize limited resources – just last night, we used the map to decide which sites to support with outreach, because we didn't have enough people to cover all 5 clinics operating today [senior program manager for a local network of clinical and community-based providers].”
		“Nice - thank you all for your hard work – we appreciate it [nurse manager within our health system]!”
	Suicide awareness	“Thanks for this important data. Keep up the hard work: you never know how this material might make a difference [physician within our health system]!”
	Tree canopy and environmental justice	“These maps are very helpful, as this weekend there’s a student-centered tree event and they were hoping to get transparencies of the various maps to print out and do overlays [representative from a school within our institution in partnership with a local high school].”
	Local health assessment indicator reports	“I've found the Local Health Assessment Indicator Reports extremely helpful in projects [health planning research analyst at local community organization].”
Suggestions for future content	**Exemplar quote**	
	More focused reviews by disease by zip codes or regions [open-ended item from website respondent 14].	
Suggestions for future content	I would love to see RocHealthData elevated. I use the data for information across the state and in some cases for data across the country. It was surprising to me to see a few Linkedin blogs about this being a new interest to organizations, like New York eHealth Collaborative when the state already had a resource that could be easily leveraged [open-ended item from website respondent 16].	
	Digital Equity Access to primary care based on different types of measures and trends [open-ended item from website respondent 37].	
	It would be great to have more zip code or census-tract-based data for the more rural counties. There is a lot of information for Monroe County, but much less for the rural areas, which is often the case across other data systems, as well [open-ended item from website respondent 29].	
Positive feedback	Current information is great. I like seeing data summaries that link with annual health observances and local health issues [open-ended item from website respondent 12].	
	I've enjoyed the variety of topics that has been provided thus far [open-ended item from website respondent 31].	
	I think the information that you are currently providing is good. Continuing to highlight areas of our community that show the health disparities and what they are would be helpful [open-ended item from website respondent 7].	
	**Theme**	**Exemplar quote**
	Praise for community engagement by RHD staff	After a segment on the local public radio station, “Thank you so much for your time and expertise [local station manager].”
		After presenting to the local health information management association (HIMA), “We enjoyed it so much and learned so much that we would like you to do a repeat performance at our annual conference for our state association [local HIMA president].”

For example, the public availability of census-tract-level COVID-19 vaccination data allowed the local health department to prioritize lower-rate tracts for the location of several vaccination clinics. Access to maps of the city of Rochester’s tree canopy helped inform a local high school’s education project about the relationship between health and environmental equity. Respiratory infection data reported during flu season was used as an educational tool by clinical nurse managers to encourage flu vaccination. Several users also shared suggestions for future content, which included a need for data on a variety of topics. Users also expressed the need for RHD to highlight disease distribution within our community with regard to health disparities.

### GA data

Table 2 shows detailed GA data, by year, and cumulatively, from January 1, 2020 through May 31, 2023. With an average of 1.40 sessions per visitor over this time period, visitors stayed an average of 1 minute and 45 s on the site, and visited 2.31 pages per session, with a bounce rate was 69.23%. However, new and returning visitors behaved differently at the site: new visitors’ session duration was 47 s, with a bounce rate of 45.5%, while those returning spent more than 4 minutes at the site, with a bounce rate of 78.5%.

**Table 2.** User engagement metrics

		January 1 through December 31, 2020	January 1 through December 31, 2021	January 1 through December 31, 2022	January 1 through May 31, 2023	January 1, 2020 through May 31, 2023
Visitors	Number of visitors	4,051	7,533	7,198	2,315	20,788
	New (%)	87.8	83.6	91.7	89.8	88.1
	Returning (%)	12.2	16.4	8.3	10.2	11.9
Sessions	Total sessions	5,730	10,977	9,151	3,204	29,062
	Sessions (per visitor)	1.41	1.46	1.27	1.38	1.40
	Session duration (mins:secs)	02:37	01:29	01:22	02:16	01:45
Pages	Total pageviews	16,641	24,491	18,371	7,708	67,211
	Pages (per session)	2.90	2.23	2.01	2.41	2.31
Bounce Rate (%)	Overall	62.06	68.23	74.89	69.32	69.23
	New users	68.98	77.49	84.80	78.94	78.50
	Returning users	46.04	48.18	40.54	45.43	45.50

**Figure 2.**
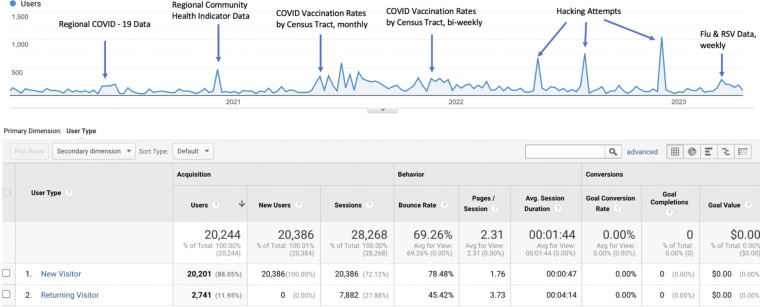
Annotated usage statistics, by visitor type, from 1/1/2019 through 5/31/2023.

Several data releases generated extensive user interest (Fig. 2), as shown by number of users related to the annotations of new or additional site events, in response to newly available local data or to an increase in frequency of those data. For example, the spike in visitors to the site in mid-2021 corresponds to the availability of local COVID-19 vaccination rate data, which was updated monthly. As these data became available bi-weekly (in late 2021), user traffic concurrently increased. Also of note in Figure 2 are the spikes in usage in late 2022. These represent hacking attempts for the site, managed effectively and minimized by the team at CARES, resulting in no damage or incursions to RHD or its members.

### Registered user data

As of May 31, 2023, RHD had 1033 registered users. Of these, 51.7% were from within the University of Rochester, 20.1% were from another educational institution, and 28.2% identified as community members. While the large majority of users (85.7%) are from the Monroe County, New York region, users are from a number of other New York counties (*n* = 17) and states other than New York (*n* = 18). Usage statistics indicate that 14.3% of users were active (logged into the site to access information) within the past three months; 18.3% of users were active within the past 6 months. Login is required to access data within the map room, however, viewing information at the site does not require a login, and thus, these percentages of user access may be higher.

The combination of qualitative feedback about how data are used, and are useful, locally (Table 1) is further reflected in the GA data on new versus returning users and the availability of local data driving site access and interest (Fig. 2). The wide regional span of our users suggest greater potential impact to be gained in the use of these clinical and SDoH data.

## Discussion

Our results emphasize the positive impact of RHD as a flexible web-based tool by both the breadth and depth of users; the data suggest that RHD is an important data resource for the upstate New York community. The engagement of returning users to the site (relative to new users) is high, shown by lower bounce rates and greater time spent at the site. User and visitor interest is driven by timely and specifically local sources of data, as shown by the increased access as local data become available, such as the availability of vaccination rate data by Monroe County census tract during the COVID-19 pandemic and the prevalence of respiratory illness in Monroe County. Specifically, our colleagues’ use of RHD shows it to be an important information source for understanding factors of vaccine hesitancy in the New York Finger Lakes region [28].

Feedback from users has been almost entirely positive. Data available at the site have been used by local academic, government, and nonprofit organizations to enact policy decisions and provide detailed information to their community constituencies. Qualitative feedback and the spike in users in response to release of local COVID-19 vaccination rate data demonstrated that the site was a powerful tool for directing resources to manage vaccine clinics where vaccinations were low.

Site maintenance and updates (of data layers and site functionality) are provided by the CARES team, with no burden on the local RHD team. The CARES team has proved particularly valuable in website management, as shown in their timely and effective intervention to manage attempted site hacks late in 2022.

There are limitations to our RHD site assessment. First, while we have many qualitative feedback points about the uses and usefulness of data provided at the site, we have relatively few data-driven such examples. We anticipate that the continuing expansion of our data sources and users will provide that data. Second, the specificity and details provided in the currently used GA package make it somewhat difficult to understand detailed user behavior at the site, such as the distinction between visitors and pageviews. The next generation of (GA4) will enable further capabilities, providing detailed information about website visitor behavior, which will allow us additional insights into our visitor and user base [29].

We will continue to provide timely and useful locally curated datasets for our users and investigate collaborative opportunities to showcase regional initiatives and health insights. While this paper has highlighted the regional and specific local data available to RHD users, it is important to note that the data layers available at the site are national, as seen in Figure 1. We seek to expand our user base and develop collaborative opportunities with other regional or national colleagues to build useful data resources within RocHealthData.org. To our knowledge, no other CTSIs have a similar, publicly accessible local data resource. We welcome the opportunity for collaboration to help us refine the translation of the SDoH into specific geographies to make available data as relevant and useful as possible.

## References

[1] Braveman P , Gottlieb L. The social determinants of health: it’s time to consider the causes of the causes. Public Health Rep. 2014;129 (Suppl 2):19–31. doi: 10.1177/00333549141291S206. PMC3863696 24385661

[2] Bacorn C , Serrano M , Koo Lin L. Review of sociodemographic risk factors for presentation with advanced non-melanoma skin cancer. Orbit. 2022;19 (5):1–6. doi: 10.1080/01676830.2022.2123930. 36120852

[3] Walker RJ , Smalls BL , Campbell JA , Strom Williams JL , Egede LE. Impact of social determinants of health on outcomes for type 2 diabetes: a systematic review. Endocrine. 2014;47 (1):29–48. doi: 10.1007/s12020-014-0195-0. 24532079 PMC7029167

[4] Chen M , Tan X , Padman R. Social determinants of health in electronic health records and their impact on analysis and risk prediction: a systematic review. J Am Med Inform Assoc. 2020;27 (11):1764–1773. doi: 10.1093/jamia/ocaa143. 33202021 PMC7671639

[5] Gu W , Hasan S , Rocca-Serra P , Satagopam VP. Road to effective data curation for translational research. Drug Discov Today. 2021;26 (3):626–630. doi: 10.1016/j.drudis.2020.12.007. 33338655

[6] Rocca-Serra P , Gu W , Ioannidis V , et al. The FAIR cookbook - the essential resource for and by FAIR doers. Sci Data. 2023;10 (1):292. doi: 10.1038/s41597-023-02166-3. 37208467 PMC10198982

[7] Xtelligent Healthcare Media. HealthITAnalytics. Tools and Strategies News. Kent J. Top 3 Data Challenges to Addressing the Social Determinants of Health. Published on February 17, 2020. https://healthitanalytics.com/news/top-3-data-challenges-to-addressing-the-social-determinants-of-health. Accessed May 17, 2023

[8] Center for Open Data Enterprise (CODE). Leveraging Data on the Social Determinants of Health. Published on December 2019. http://reports.opendataenterprise.org/Leveraging-Data-on-SDOH-Summary-Report-FINAL.pdf. Accessed June 14, 2023

[9] Highberger JP , Merriman-Nai S. The value (and nuances) of mapping as a public health tool. Dela J Public Health. 2021;7 (3):6–9. doi: 10.32481/djph.2021.07.003. PMC8352412 34467203

[10] Mittelstadt BD , Floridi L. The ethics of big data: current and foreseeable issues in biomedical contexts. Sci Eng Ethics. 2016;22 (2):303–341. doi: 10.1007/s11948-015-9652-2. 26002496

[11] Kim J , Kim DH , Lee J , Cheon Y , Yoo S. A scoping review of qualitative geographic information systems in studies addressing health issues. Soc Sci Med. 2022;314:115472. doi: 10.1016/j.socscimed.2022.115472. 36334495

[12] Highfield L , Arthasarnprasit J , Ottenweller CA , et al. Interactive web-based mapping: bridging technology and data for health. Int J Health Geogr. 2011;10 (1):69. doi: 10.1186/1476-072X-10-69. 22195603 PMC3258200

[13] RocHealthData. Community Health Needs Assessment. https://rochealthdata.org/rochester-area-community-health-needs-assessment-tool/. Accessed December 20, 2023.

[14] RocHealthData. Community Health Indicators. https://rochealthdata.org/2020-chi/. Accessed June 14, 2023.

[15] RocHealthData. COVID-19 Vaccination Rates for the City of Rochester. Last updated on October 6, 2022. https://rochealthdata.org/covid-19-vaccination-rates/. Accessed June 14, 2023.

[16] RocHealthData. Monroe County Influenza & RSV Surveillance. October 1, 2022 – April 29, 2023. https://rochealthdata.org/influenza-surveillance-dashboard/?l=36055. Accessed June 14, 2023.

[17] RocHealthData. Improving Reading Levels: Boys & Girls Clubs of Rochester. Published on April 18, 2022. https://rochealthdata.org/2022/04/18/improving-reading-levels-boys-girls-clubs-of-rochester/. Accessed June 14, 2023.

[18] RocHealthData. May is Older Americans Month: Poverty in Later Life. Published on May 24, 2021. https://rochealthdata.org/2021/05/24/may-is-older-americans-month-poverty-in-later-life/. Accessed June 14, 2023.

[19] RocHealthData. Air Quality: Current Wildfire and Smoke. Published on June 6, 2023. https://rochealthdata.org/2023/06/06/air-quality-current-wildfire-and-wildfire-smoke/. Accessed June 14, 2023.

[20] RocHealthData. World Rivers Day: Seneca Park Zoo’s Work to Conserve the Lake Sturgeon. Published on September 21, 2021. https://rochealthdata.org/2021/09/21/world-rivers-day-seneca-park-zoos-work-to-conserve-the-lake-sturgeon/. Accessed June 14, 2023.

[21] Google analytics. Published in 2023. https://analytics.google.com/analytics/web/. Accessed June 28, 2023.

[22] Budd BQ. Website data and uses for strategic marketing-a commercial experience. Int J Manag Inf Syst. 2012;16 (3):239–246. doi: 10.19030/ijmis.v16i3.7076.

[23] Fang W. Using google analytics for improving library website content and design, a case study. Libr Philos Pract. 2007;121:1–17.

[24] Jansen BJ , Jung S , Salminen J. Measuring user interactions with websites: a comparison of two industry standard analytics approaches using data of 86 websites. PloS One. 2022;17 (5):e0268212. doi: 10.1371/journal.pone.0268212. 35622858 PMC9140287

[25] Fundingsland EL , Fike J , Calvano J , Beach J , Lai D , He S. Methodological guidelines for systematic assessments of health care websites using web analytics: tutorial. J Med Int Res. 2022;24 (4):e28291. doi: 10.2196/28291. PMC9055485 35436216

[26] Gale NK , Heath G , Cameron E , et al. Using the framework method for the analysis of qualitative data in multi-disciplinary health research. Bmc Med Res Methodol. 2013;13 (1):117. doi: 10.1186/1471-2288-13-117. 24047204 PMC3848812

[27] Creamer EG. An Introduction to Fully Integrated Mixed Methods Research. Thousand Oaks, CA: SAGE; 2018.

[28] Nooraie RY , Reichelt M , Dadgostar P , et al. The pragmatic, rapid, and iterative dissemination and implementation (PRIDI) framework to fit implementation activities to shifting landscapes; the case of an intervention to address COVID-19 vaccine hesitancy. BMC Public Health. 2023;19:110.

[29] Google Analytics. Analytics Help. [GA4] Introducing the next generation of Analytics, Google Analytics 4. https://support.google.com/analytics/answer/10089681?hl=en. Accessed June 15, 2023.

